# KDM3A drives NSCLC proliferation and metastasis via H3K9 demethylation, EMT activation, and MMP-9 upregulation

**DOI:** 10.17305/bb.2025.11251

**Published:** 2025-11-03

**Authors:** Bingqing Shi, Zhe Wang, Lei Xiu, Luyao Li, Xiaolian Yang, Guanhua Wang, Jianjun Li, Hu Wang, Yuning Han

**Affiliations:** 1 Department of Cardio-Thoracic Surgery, General Hospital of Ningxia Medical University, Yinchuan, China; 2 Department of General Thoracic Surgery, General Hospital of Ningxia Medical University, Yinchuan, China; 3 Anesthesiology Department, Yinchuan Hospital of Traditional Chinese Medicine, Yinchuan, China

**Keywords:** Demethylation, non-small cell lung cancer, epithelial–mesenchymal transition, cell invasion, cell proliferation, KDM3A

## Abstract

Histone methylation dysregulation is a crucial epigenetic driver of lung carcinogenesis; however, the role of lysine-specific demethylase 3A (KDM3A) in non-small cell lung cancer (NSCLC) remains inadequately understood. In this study, we established NSCLC cell models with both KDM3A overexpression and knockdown to investigate its functional impact. *In vitro* assays demonstrated that KDM3A depletion increased histone H3 lysine 9 dimethylation (H3K9me2), suppressed cell proliferation, and impaired migration and invasion by attenuating epithelial–mesenchymal transition (EMT) and the expression of matrix metalloproteinase-9 (MMP-9). Conversely, KDM3A overexpression led to reduced H3K9me2 levels, activated EMT, and enhanced metastatic potential. Mechanistically, KDM3A decreased H3K9me2 occupancy at the promoters of *VIM* and *MMP-9*, thus upregulating their expression. Additionally, KDM3A downregulated E-cadherin by activating the p-STAT3 pathway. *In vivo*, KDM3A knockdown significantly inhibited tumor growth in xenograft models. Clinical analyses revealed elevated KDM3A expression in metastatic NSCLC tissues, with a negative correlation between KDM3A and H3K9me2, and a positive association between KDM3A and FOXP3. These findings establish KDM3A as an epigenetic modulator of NSCLC progression through H3K9me2-dependent regulation of EMT and metastatic pathways, highlighting its therapeutic potential for NSCLC treatment.

## Introduction

Lung cancer is the leading cause of cancer-related mortality globally and is primarily classified into non-small cell lung cancer (NSCLC) and small cell lung cancer (SCLC). Despite advancements in lung cancer therapies, NSCLC constitutes over 75% of all lung cancer cases. A comprehensive understanding of the molecular mechanisms underlying NSCLC is essential for developing more effective prevention and treatment strategies.

Lung cancer is the most prevalent malignant tumor, with NSCLC accounting for 80%–85% of all cases [1, 2]. Investigating the mechanisms and therapeutic strategies for lung cancer progression remains a critical concern. For early diagnosis of NSCLC and the development of tailored therapies, a thorough examination of potential disease indicators and targetable molecules is necessary. Recent advancements in epigenetic research have enhanced our understanding of lung cancer pathogenesis and facilitated personalized treatment approaches [3, 4]. Epigenetic mechanisms, including post-translational modifications of DNA and histones that affect chromatin structure, regulate gene expression during normal development and contribute to carcinogenesis and cancer progression [5, 6].

In eukaryotic cells, the epigenetic regulation of gene expression primarily involves post-translational modifications of the N-terminal tails of core histones and DNA methylation of CpG islands. The nature and genomic location of these modifications directly influence chromatin accessibility and transcriptional activity [7–9]. Histone methylation, a rapidly evolving area of research, plays a pivotal role in regulating transcription [10].

KDM3A is an epigenetic activator that regulates gene expression by demethylating H3K9me2 modifications. Although KDM3A has been implicated in tumor progression across various cancers, its specific function and molecular mechanisms in NSCLC require further investigation. The methylation of histone H3 lysine 9 (H3K9) is a critical epigenetic mark necessary for heterochromatin formation and the regulation of diverse biological processes, including gene silencing [11, 12]. As a demethylase of H3K9me2, KDM3A can significantly influence cellular functions by dysregulating gene expression programs [13–15]. Genetic ablation or mis-targeting of a single H3K9 methyltransferase can result in impaired cell differentiation, loss of tissue properties, premature senescence, and cancer [16]. Supporting its pro-tumorigenic role [17], KDM3A knockdown has been shown to suppress the invasive and migratory capacities of cancer cells in various contexts, including breast cancer [18], abdominal aortic aneurysms [15], clear cell renal cell carcinoma [18], abdominal aortic tumors [19], and glioblastoma [20, 21].

However, a comprehensive understanding of the role of the H3K9 methylation regulator KDM3A in NSCLC, particularly its underlying molecular mechanisms, remains to be explored.

In this study, we investigated the role of KDM3A in the proliferation and migration of NSCLC cells both *in vitro* and *in vivo*. We assessed the impact of KDM3A on H3K9me2 levels via Western blot analysis. Furthermore, we conducted a detailed examination of how KDM3A influences genes associated with cell migration and invasion using a combination of techniques, including Western blot, qRT-PCR, chromatin immunoprecipitation (ChIP), wound healing, transwell assays, and xenograft models. Finally, we evaluated the expression of KDM3A in clinical NSCLC samples through immunohistochemistry (IHC) staining. These findings provide new insights into KDM3A as a potential target for NSCLC therapy and underscore the importance of further investigating its role in tumor progression.

## Materials and methods

### Cell culture

The NSCLC cell lines A549, H1299, and HCC827 used in this investigation were obtained from ATCC (Manassas, VA, USA). Cells were authenticated and tested for mycoplasma contamination using a PCR kit (Beyotime Biotechnology Co., Cat# C0301S, Shanghai, China). H1299 and HCC827 cells were cultured in Roswell Park Memorial Institute 1640 (RPMI-1640) medium (Gibco, C11875500BT, Beijing, China), while A549 cells were grown in Dulbecco’s Modified Eagle Medium (DMEM) with elevated glucose (Hyclone, SH30284.01, Shanghai, China), supplemented with 10% fetal bovine serum (FBS) (Cytiva, SH30406.05, Tauranga, New Zealand) and 100 U/mL Penicillin–Streptomycin Solution (Gibco, 10378016, CA, USA). The histone demethylase inhibitor IOX1 was obtained from Medchemexpress (Cat# HY-12304). CBA-1 was sourced from ProbeChem (Cat# PC-20353), and cisplatin was acquired from Medchemexpress (Cat# HY-17394).

### Quantitively reverse transcription PCR (qRT-PCR)

Standard techniques with Trizol reagents (Sigma-Aldrich; T9424; Germany) were used to extract RNA from cells. Following a 10-min lysing period and a 15-min centrifugation at 12,000 *g*, the samples were exposed to isopropanol treatment. After that, the extracted RNA was reverse transcribed into complementary DNA (cDNA) through the PrimeScript RT kit (Takara Holdings Inc; RR047Q; Kyoto, Japan). qRT-PCR was carried out using the TB Green@Premix Ex Taq™ II (Takara; CN830S; Beijing, China) guidelines using an Applied Biosystems, Inc. (Carlsbad, CA, USA) 7500 system. The primers in both forward and reverse for human *KDM3A* were 5′- GCCAACATTGGAGACCACTTCTG-3′ and 5′- CTCGAACACCTTTGACAGCTCG-3′. Human E-cadherin (*CDH1*), forward: 5′- GCCTCCTGAAAAGAGAGTGGAAG-3′, reverse: 5′- TGGCAGTGTCTCTCCAAATCCG-3′, human Vimentin (*VIM*), forward: 5′-AGGCAAAGCAGGAGTCCACTGAA-3′, reverse: 5′- ATCTGGCGTTCCAGGGACTCAT-3′, human *MMP9*, forward: 5′- GCCACTACTGTGCCTTTGAGTC-3′, reverse: 5′- CCCTCAGAGAATCGCCAGTACT-3′, human *KDM3B*, forward: 5′- GCTCGTAATGTCTGAGAAGGAGG-3′, reverse: 5′- CACATTTGCGACAAACCCAGTGG-3′; human *KDM3C*, forward: 5′- TCCTGTCAGACCTTCCAGTGCA-3′, reverse: 5′-GTGGATGCAACAGACCGTAATGG-3′, human Actin, forward: 5′- CACCATTGGCAATGAGCGGTTC-3′, reverse: 5′- AGGTCTTTGCGGATGTCCACGT-3′.

### Western blot

Total protein was extracted from samples using RIPA lysis buffer (Beyotime Biotechnology Co., G3424; Shanghai, China). The Pierce™ BCA Protein Assay Kit (Thermo Fisher, Cat#23227) was employed to quantify the protein concentrations. Proteins were separated by SDS-PAGE electrophoresis and subsequently transferred to a polyvinylidene fluoride (PVDF) membrane (Biorad, 1620177, Shanghai, China). After blocking the membranes with 5% non-fat milk at room temperature for one hour, the membranes were washed three times with 1 × TBST (Tris: 20 mM, NaCl: 150 mM, Tween^®^ 20: 0.1% (w/v)). Membranes were then incubated overnight at 4 ^∘^C with antibodies against KDM3A (GeneTex, 54313, 1:1000), KDM3B (Proteintech, 19915-1-AP, 1:1000), H3K9me2 (Cell Signaling Technology, 4658T, 1:2000), β-actin (Santa Cruz Biotechnology, sc-47778, 1:1000), matrix metalloproteinase-9 (MMP-9) (Santa Cruz Biotechnology, sc-393859, 1:1000), E-cadherin (Santa Cruz Biotechnology, sc-8426, 1:1000), Vimentin (Santa Cruz Biotechnology, sc-6260, 1:1000), FAK (Cell Signaling Technology, 3285S, 1:1000), pFAK (Tyr397) (Cell Signaling Technology, 3283, 1:1000), STAT3 (Cell Signaling Technology, 4904, 1:1000), pSTAT3 (Y705) (Cell Signaling Technology, 9131, 1:1000), and GAPDH (Proteintech, HRP-60004, 1:5000). Membranes were then washed three times with 1 × TBST and incubated for one hour at room temperature with HRP-labeled secondary antibodies (ab205718 for anti-Rabbit, ab20571 for anti-Mouse, Abcam, 1:10000). Protein bands were visualized using Western ECL substrate (Biorad; Cat# 1705061) and imaged with a Bio-Rad Imaging system (ChemiDoc, Bio-Rad, CA, USA). ImageJ software (NIH, Bethesda, MD, USA) was utilized for quantitative analysis, with β-actin and GAPDH serving as internal loading controls.

### Plasmid construction

The full-length FLAG-tagged *KDM3A* was amplified from H1299 cDNA using the following primers: forward primer 5′-CCGGGTACCATGGACTACAAAGACGATGACGACAAGTGCTCACGCTCGGAGAA-3′ and reverse primer 5′-CCGCTCGAGTGCCTGAAGAGTTTGAACAGCTGCCT-3′. The Kpn I and Xho I-digested fragment was purified and ligated into a linearized pcDNA3.1 vector. Positive plasmids were confirmed by Sanger sequencing. *KDM3A* shRNA constructs were integrated into pLKO.1 vectors, with the target sequence for *KDM3A* shRNA being 5′-CTGAAGGTGTGTGTGGAATTT-3′ and the non-target control sequence being 5′-TCTCGCTTGGGCGAGAGTAAG-3′.

### Cell transfection

Cells (5 × 10^5^) were seeded in 6-well plates. After 12 h, siRNA was transfected into the cells using Lipofectamine RNAiMAX transfection reagent (Invitrogen; 13778100; USA). The target sequences for *KDM3A* siRNA1 and siRNA2 were GAAGGCTTCTTAACACCAA and GAAATCAACTACTGTACAA, respectively. The non-target siRNA was procured from Santa Cruz Biotechnology (Cat#: sc-44236, CA, USA). KDM3A overexpression or shRNA plasmids were transfected into cells using Lipo3000 reagent (Invitrogen; L3000075; USA). Control cells were transfected with empty pcDNA3.1 or pLKO.1 plasmids. A 1% GFP plasmid was co-transfected to visualize transfection efficiency. Stable KDM3A knockdown cell lines were selected by treating with puromycin (Invivogen, ant-pr-1, CA, USA) at 5 µg/mL for 3 days. Puromycin-resistant cells were then seeded in 10 cm dishes for single-clone selection, and the KDM3A knockdown cell lines were subsequently utilized for further studies.

### Wound healing assay

For knockdown or overexpression experiments, 6-well plates were used to seed cells at a density of 5 × 10^5^ cells per well. After 24 h, when the cells had reached approximately 100% confluence, a sterile 200 µL pipette tip was used to scrape down the midline of the well. The cells were washed twice with PBS, serum-free medium was added, and images were captured at specified time points using a Nikon Ti2-E microscope (Nikon Instrument Inc., Tokyo, Japan). For inhibitor experiments, cells were seeded similarly and then scraped and washed as described. Serum-free medium containing various inhibitors was added, and images were taken at designated times. Migration distances were analyzed using ImageJ software (NIH, Bethesda, MD, USA).

### Cell proliferation, viability, and invasion assays

For cell proliferation assays, after transfection with siRNA or plasmids for 24 h, cells were seeded at a density of 1 × 10^5^ cells per well in a 6-well plate. Following 72 h, cells were harvested and quantified using the Cellmeter Spectrum (Nexcellom Bioscience, USA). For viability testing, 3000 cells were seeded in a 96-well plate. After 24 h, cells were treated with inhibitors for either 72 h or 24 h (for cisplatin combination treatment). Cell viability was assessed using a CCK-8 kit (Beyotime, C0038, Shanghai, China) and measured with a Tecan Spark plate reader (Tecan Trading AG, Switzerland), with five wells per group evaluated. Cell invasion assays were conducted using an 8 µm pore size. The invasion chambers were coated with Matrigel (Corning; 354481; USA). For cells transfected with plasmids, 24 h post-transfection, cells were detached using a 0.25% trypsin-EDTA solution (Coolaber; SL6020-500 mL; Beijing, China) and seeded in the upper chamber at a density of 2 × 10^4^ cells per well. Cell culture medium without FBS was added to the upper chamber, while medium with FBS was added to the lower chamber. In the inhibitor treatment groups, cells were pretreated with inhibitors for 48 h prior to seeding. In the upper chamber, medium with inhibitors but no FBS was added, while the lower chamber contained both inhibitors and FBS. After 20 h, non-invading cells in the upper layer were gently removed using a cotton swab. Cells that invaded to the bottom layer were fixed and stained with 1% crystal violet (Beyotime; C0121-100ml; China). Images were taken using a Nikon Ti2-E microscope (Nikon Instrument Inc., Ti2-E, Tokyo, Japan) and cell counts were performed for five fields per sample using ImageJ software.

### Colony formation experiments

NSCLC cells were cultivated in 6-well plates at a concentration of 500 cells per well for eight days. Following cultivation, the cells were washed twice with PBS, fixed with 4% paraformaldehyde (Ansiang; L-AX2356; Beijing, China), and stained with a crystal violet staining solution (Beyotime; C0121-100 mL; China). After staining, the cell colonies were photographed using a Bio-Rad imaging system (ChemiDoc, Bio-Rad, CA, USA).

### Chromatin immunoprecipitation

Chromatin immunoprecipitation was conducted in accordance with the instructions provided in the ChIP Histone H3 [Dimethyl Lys9] Kit (Novus, NBP1-71712). Briefly, cells were crosslinked with 1% formaldehyde for 10 min at room temperature, quenched with 125 mM glycine, and lysed in RIPA lysis buffer. Chromatin was sonicated to an average size of 200–1000 bp. After pre-clearing with Protein A/G beads, the lysates were incubated overnight at 4 ^∘^C with an anti-H3K9me2 antibody or an IgG control. Immune complexes were captured with Protein A/G beads, washed sequentially with low-salt, high-salt, LiCl, and TE buffers, and then eluted in ChIP elution buffer. Crosslinks were reversed at 95 ^∘^C for 10 min, and DNA was purified. Quantitative PCR (qPCR) was performed using SYBR Green Master Mix (Bio-rad, Cat#1725121, USA) on an Applied Biosystems (Carlsbad, CA, USA). Primer sequences using is listed below: *CDH1*-forward: 5′-GAACCCTCAGCCAATCAGC-3′, reverse: 5′-CTGACTTCCGCAAGCTCACA-3′. *VIM*- forward: 5′-GAGGGGACCCTCTTTCCTAA-3′, reverse: 5′-GAGAGTGGCAGAGGACTGGA -3′. *MMP9*-forward: 5′-TCACAGGAGCGCCTCCTTAA-3′, reverse: 5′-AGCAAAGCAGCAGCCCAGCA-3′. Enrichment was calculated as % input (2ˆ (ΔCt [Input-ChIP])) and normalized to IgG controls.

### *In vivo* Xenograft model

The methods used for conducting animal research were authorized by the Ningxia Medical University Animal Care and Use Committee (KYLL-2024-0794). Nude mice (Gempharmatech Company, 20–22 g, 6–8 weeks of age, female: male = 1:1) were utilized for xenograft experiments. All animal protocols were conducted in the animal facility at Ningxia Medical University in accordance with federal, local, and institutional guidelines. A suspension of 5 × 10^6^ H1299 cells in RPMI 1640 medium was injected subcutaneously into nude mice. The volume and body weight of the mice were recorded, and tumor volumes were measured and calculated using the formula [0.5 × L (long dimension) × W^2^ (short dimension)] twice a week. The mice were sacrificed via CO_2_ inhalation approximately three weeks after the injection. The tumors were excised, weighed, and retained for hematoxylin–eosin (HE) staining and IHC staining.

### Ethic and clinical sample collection

Clinical samples were collected from a total of 30 lung adenocarcinoma patients who underwent surgical resection between 2023 and 2024 without prior chemotherapy or radiation therapy. Clinical characteristics of the patients and tumor as well as adjacent tumor samples were collected.

### HE and IHC staining

Tumor tissues were fixed in formalin solution immediately after harvesting. The tissues were dehydrated with ethanol, embedded in paraffin, and sectioned onto slides for the staining procedure. HE staining was performed following the guidelines of the HE stains solution kit (Applygen, C1410, Beijing, China). Briefly, slides were deparaffinized and rehydrated by immersing them in xylene, 100% ethanol, 95% ethanol, and 80% ethanol for 5 min each. The slides were then placed in deionized H_2_O three times for 3 min each. The deparaffinized slides were stained with hematoxylin for 3 min and subsequently washed with deionized H_2_O. Following this, the slides were washed with hydrochloric acid ethanol and deionized H_2_O. Finally, the slides were stained with eosin for 2 min and washed with deionized H_2_O. IHC staining was performed following the protocol provided by Elabscience (Cat#: E-IR-R220, Wuhan, China). Briefly, slides underwent dewaxing and antigen retrieval using the Dewaxing/Antigen Retrieval Buffer provided in the kit in a pressure cooker for 20 min. After drying, the slides were incubated with SP reagent B at room temperature for 15 min. After washing with TBS three times for 2 min each, the slides were stained with primary antibodies (KDM3A (1:100), H3K9me2 (4658T, 1:200), Ki67 (Cell Signaling Technology, 9129T, 1:500, USA), CD34 (Abcam, ab81289, 1:200, USA), FOXP3 (Abcam, ab20034, 1:500, USA) overnight at 4 ^∘^C. Following three washes with TBS, slides were stained with HRP-labeled secondary antibody for 1 h at room temperature. Positive signals were visualized by adding the DAB buffer provided in the kit. All the slides were dehydrated by 85% ethanol, 95% ethanol ′2, absolute ethanol ′2, 3min each. The slides were then transparentized with xylene and sealed with neutral gum. Imaging was performed using a Zeiss Axio Imager M2 microscope (Carl Zeiss, Oberkochen, Germany). For clinical slides, KDM3A, H3K9me2, and FOXP3 IHC staining were scored by a pathologist as 0 (no positive staining), 1+ (< 15%), 2+ (15%–30%), and 3+ (> 30% of the cells stained positive). Spearman correlation analysis was utilized to evaluate the correlation between the expression of the three proteins.

### Ethical statement

This study was approved by the Research Ethics Committee of Ningxia Medical University, which authorized the methods used for conducting animal research and patient sample collection with informed consent (Approved number: KYLL-2024-0794).

### Statistical analysis

Images were analyzed using ImageJ software. Data analysis was conducted using GraphPad Prism 8.02 (GraphPad, La Jolla, CA, USA). Results from three separate experiments are presented as mean ± standard deviation (SD). SD was calculated by determining the squared differences of each data point from the mean, summing these squared differences to obtain the variance, and then taking the square root of the variance. A Student’s *t*-test was employed to evaluate differences between groups. Spearman correlation analysis was utilized to assess the correlation between protein expression in clinical samples. A *P* value of <0.05 was considered statistically significant.

## Results

### Knockdown of KDM3A inhibited cell proliferation and migration

**Figure 1.**
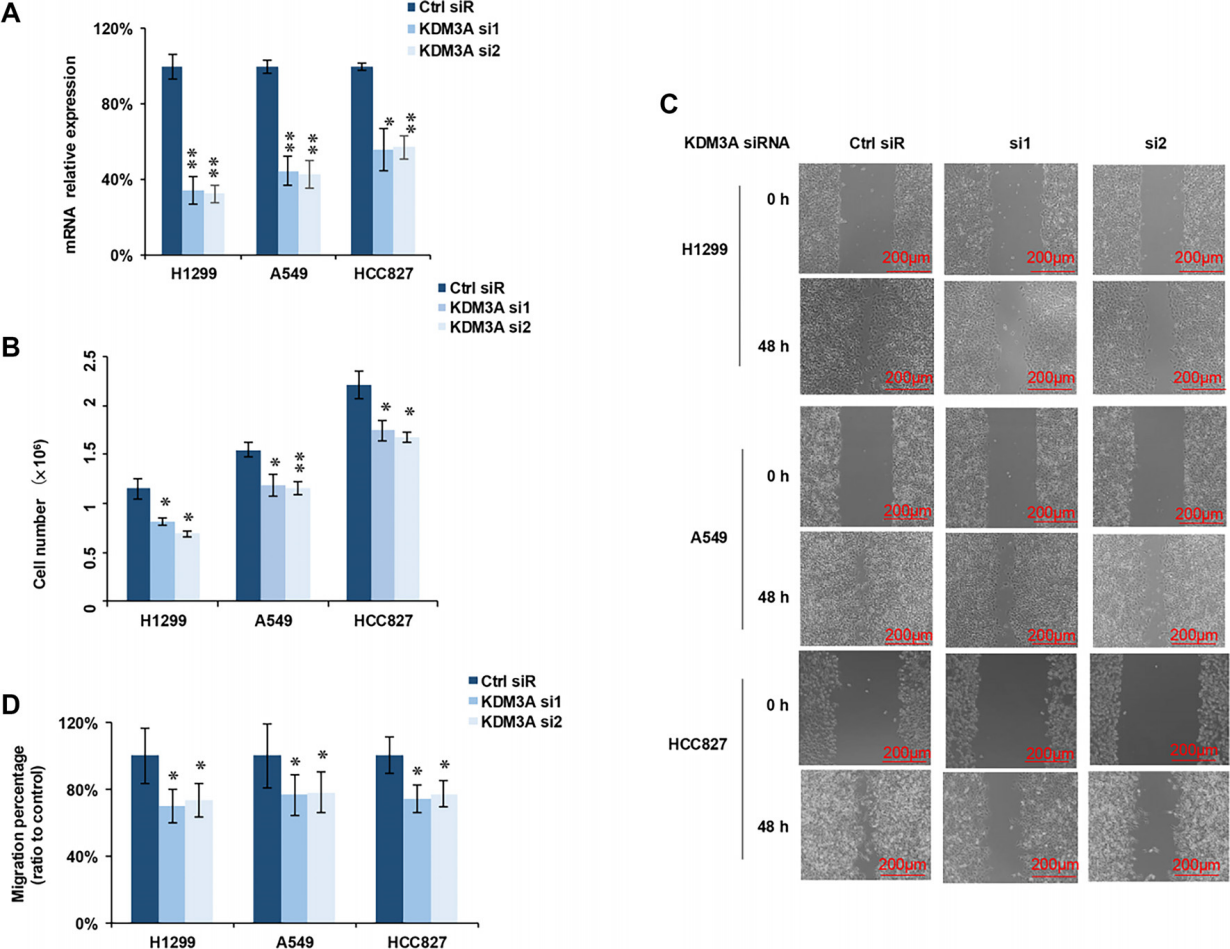
**KDM3A knockdown inhibits cell proliferation and migration.** (A) Two specific KDM3A siRNAs significantly downregulated KDM3A mRNA expression across all three cell lines (*n* ═ 3). (B) KDM3A knockdown markedly reduced cell proliferation in all three cell lines (*n* ═ 3). (C) Representative images of wound-healing assays in H1299, A549 and HCC827 cells transfected with control siRNA (Ctrl siR) or KDM3A siRNAs (si1, si2) at 0 h and 48 h. Scale bar, 200 µm. (D) Quantification of cell migration in the wound-healing assay; migration is expressed as migration percentage relative to the Ctrl siR group (*n* ═ 6). All statistical analyses were performed using Student's *t*-test, with significance levels indicated as follows: *p < 0.05; **p < 0.01; ***p < 0.001 ns: no significant differences. Abbreviations: KDM3A: Lysine-specific demethylase 3A; siRNA: Small interfering RNA; H1299/A549/HCC827: NSCLC cell lines.

KDM3A facilitates the enzymatic removal of methyl groups from the transcriptionally repressive histone mark H3K9me2. By erasing these repressive marks, it promotes a more open chromatin state, thereby modulating transcriptional activation [22--25]. To investigate the functional impact of KDM3A on NSCLC, we knocked down its expression using siRNAs (Figure 1A). Subsequent evaluation of cell proliferation in three cancer cell lines (H1299, A549, and HCC827) revealed that KDM3A knockdown significantly diminished proliferation in all cell lines compared to the control group (Figure 1B). Furthermore, the wound-healing assay demonstrated a significant reduction in cancer cell migration (Figure 1C) following KDM3A knockdown in H1299, A549, and HCC827 cell lines (Figure 1D). These preliminary results indicate that KDM3A knockdown hinders both tumor cell proliferation and migration in NSCLC.

### Knockdown of KDM3A increases H3K9me2 and inhibits EMT-related signaling

**Figure 2.**
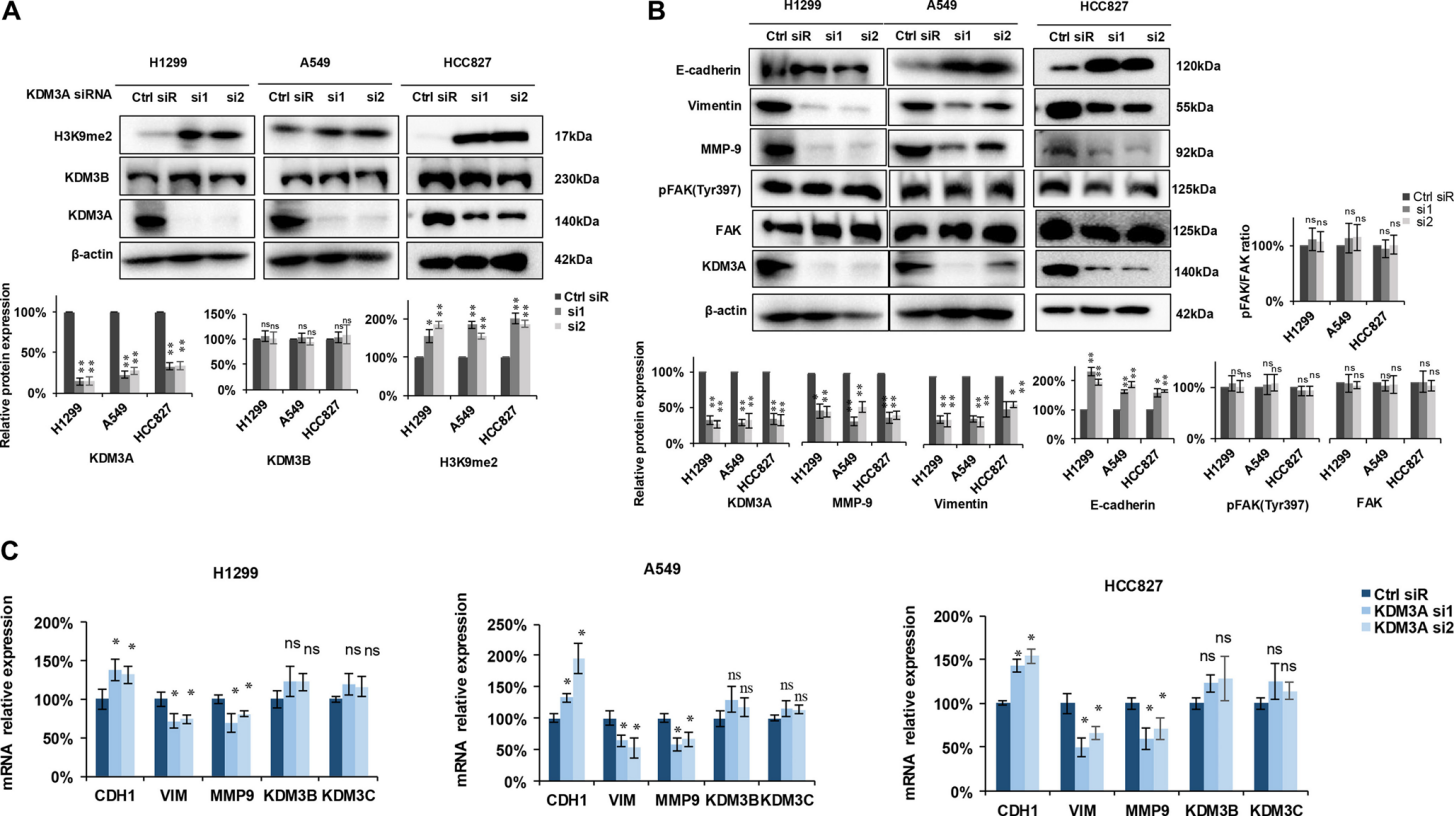
**Knockdown of KDM3A enhances H3K9me2 and modulates the cell invasion signaling pathway.** (A) Two siRNAs inhibited KDM3A expression, resulting in an upregulation of H3K9me2 across the three cell lines, with no significant changes observed in KDM3B expression. The upper panel presents representative Western blot images, while the lower panel displays statistical analyses of the Western blot data (*n* ═ 3). (B) The expression levels of E-cadherin, vimentin, MMP-9, pFAK, and FAK were evaluated using Western blotting. The upper panel shows representative Western blot images, and the lower panel provides statistical analyses (*n* ═ 3). The ratio of pFAK to FAK was calculated after normalization to the β-actin loading control. (C) The expression levels of E-cadherin, vimentin, MMP-9, KDM3B, and KDM3C were assessed using qRT-PCR (*n* ═ 3). All experiments were conducted a minimum of three times. Statistical analyses were performed using the Student's *t*-test, with significance levels indicated as follows: *p < 0.05; **p < 0.01; ***p < 0.001, ns: no significant differences. Abbreviations: KDM3A: Lysine-specific demethylase 3A; H3K9me2: Histone H3 lysine 9 dimethylation; KDM3B: Lysine-specific demethylase 3B; KDM3C: Lysine-specific demethylase 3C; siRNA: Small interfering RNA; FAK: Focal adhesion kinase; H1299/A549/HCC827: NSCLC cell lines; VIM: Vimentin.

To explore the mechanism by which KDM3A regulates migration in NSCLC, we evaluated metastasis-related proteins by Western blot analysis. As shown in Figure 2A (upper and lower panels), KDM3A knockdown significantly increased H3K9me2 levels in both siRNA groups (si1 and si2) compared to the control group (Ctrl siR) across H1299, A549, and HCC827 cell lines, without affecting KDM3B expression. E-cadherin protein levels increased, while MMP-9 and Vimentin levels decreased in the KDM3A knockdown groups (si1 and si2) compared to the control group (Ctrl siR). In contrast, the expression of FAK, phosphorylated FAK (Tyr397), and the pFAK/FAK ratio remained unchanged (Figure 2B). Quantification confirmed a significant increase in E-cadherin and H3K9me2, along with a significant decrease in MMP-9 and Vimentin following KDM3A knockdown, while no significant changes were observed in pFAK, FAK, or the pFAK/FAK ratio (Figure 2B, lower and right panels). We next used qRT-PCR to assess the mRNA expression of EMT-related genes and other KDMs. Consistent with the protein data, *CDH1* (E-cadherin) mRNA was significantly upregulated in the si1 and si2 groups, while the mRNA expression of VIM and MMP-9 decreased. However, no significant differences were observed in the mRNA expression of *KDM3B* or *KDM3C* (Figure 2C). Collectively, these results demonstrate that KDM3A knockdown upregulates H3K9me2 and suppresses the expression of key genes (VIM, MMP-9) associated with cell invasion, indicating that KDM3A promotes tumor cell invasion in a H3K9me2-dependent manner.

### Overexpression of KDM3A decreases H3K9me2 levels and promotes EMT-related signaling

**Figure 3.**
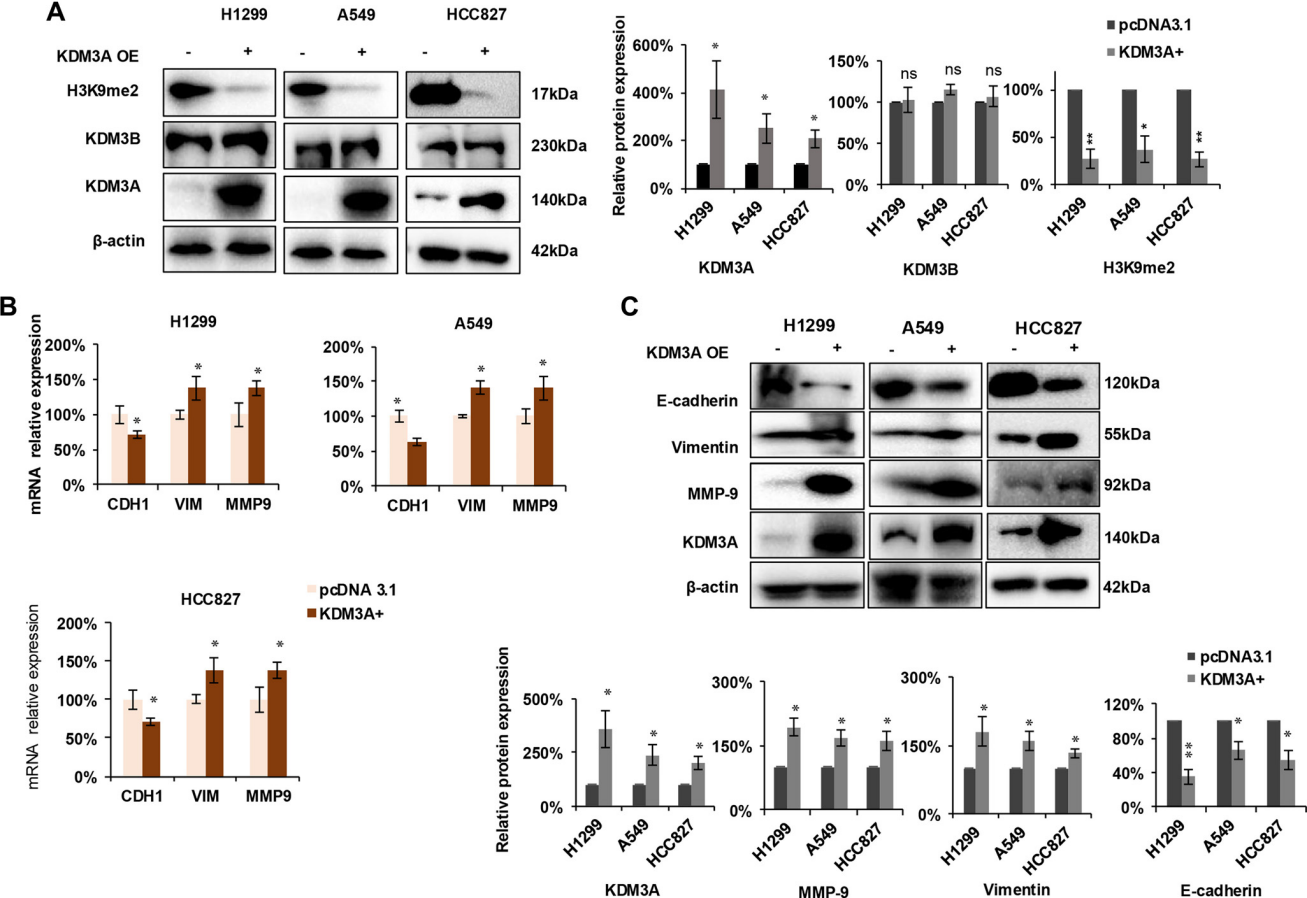
**KDM3A overexpression upregulates EMT and MMP-9 by regulating H3K9me2 modification of VIM and MMP-9.** (A) H3K9me2 expression decreased following KDM3A overexpression in three cell lines. Left panel: representative Western blot images; right panel: statistical analysis for Western blot (*n* ═ 3). (B) The expression levels of MMP-9, E-cadherin, and vimentin were evaluated using qRT-PCR across three cell lines (*n* ═ 3). (C) The expression of MMP-9, E-cadherin, and vimentin was analyzed through Western blotting. Upper panel: representative Western blot images; lower panel: statistical analysis for Western blot (*n* ═ 3). All statistical analyses were performed using Student's *t*-test. Significance levels are indicated as follows: *p < 0.05; **p < 0.01; ***p < 0.001, ns: no significant differences. Abbreviations: KDM3A: Lysine-specific demethylase 3A; EMT: Epithelial-mesenchymal transition; H3K9me2: Histone H3 lysine 9 dimethylation; VIM: Vimentin; MMP-9/MMP9: Matrix metalloproteinase-9; E-cadherin: Epithelial cadherin.

Following the confirmation that KDM3A knockdown impedes the growth and invasion of NSCLC cells, we investigated the functional consequences of KDM3A overexpression. We overexpressed KDM3A in H1299, A549, and HCC827 cell lines and analyzed key markers using Western blotting. KDM3A overexpression significantly decreased H3K9me2 levels across all three cell lines without altering KDM3B expression, compared to the control group (Figure 3A, left and right panels). Additionally, KDM3A overexpression led to a substantial reduction in E-cadherin expression and an elevation in Vimentin and MMP-9 expression at both the mRNA level (Figure 3B) and protein level (Figure 3C).

**Figure 4.**
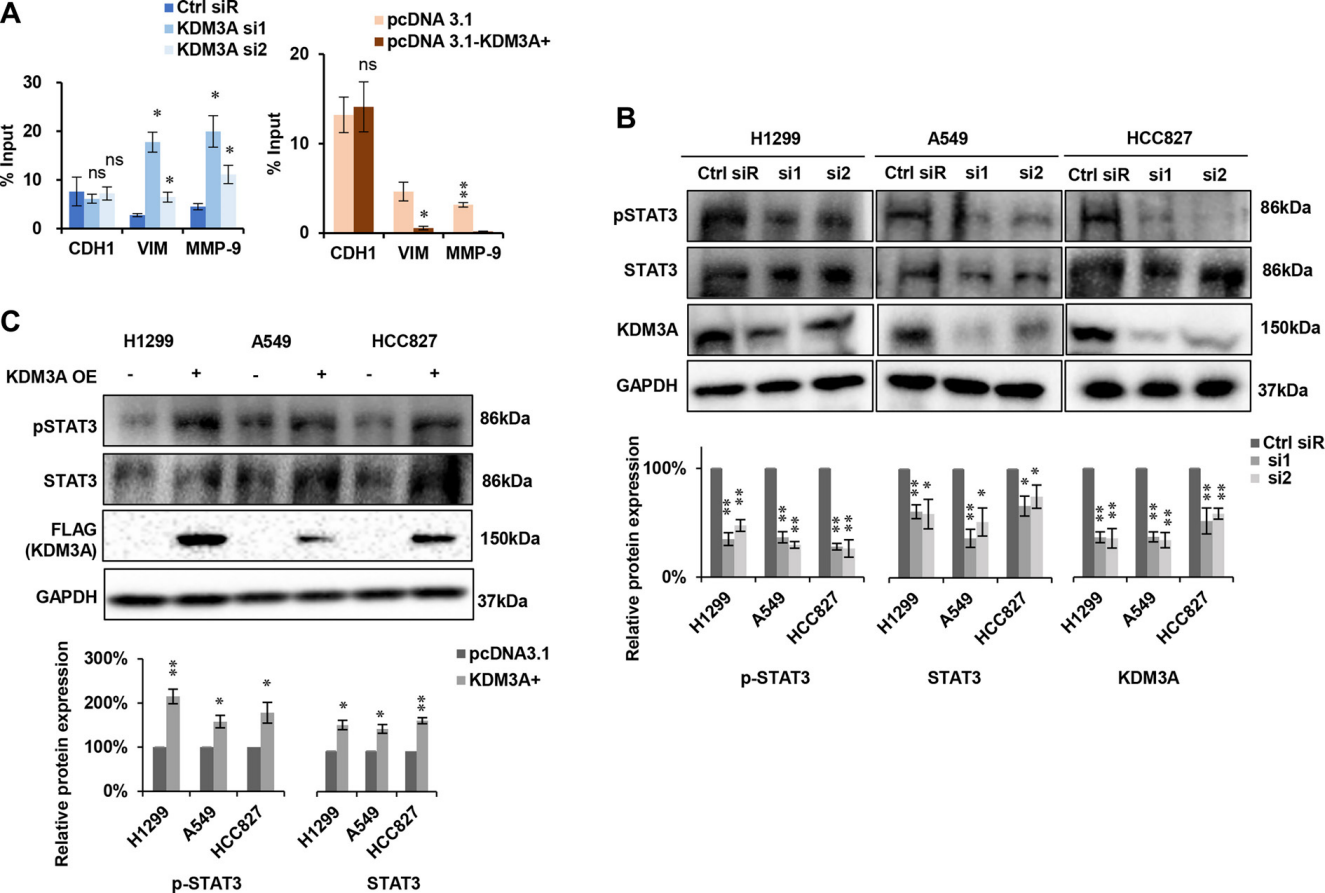
(A) Modulation of KDM3A-regulated H3K9me2 occupancy on the promoters of VIM and MMP-9, with no effect on CDH1. Left panel: Knockdown of KDM3A resulted in the upregulation of H3K9me2 at VIM and MMP-9, with no effect on CDH1. Right panel: Overexpression of KDM3A led to the downregulation of H3K9me2 occupancy at VIM and MMP-9, while CDH1 remained unaffected (*n* ═ 3). (B) The expression levels of pSTAT3 and STAT3 following KDM3A knockdown were assessed by western blotting. Upper panel: Representative western blot images; lower panel: Statistical analysis of western blot results (*n* ═ 3). (C) The protein expression levels of pSTAT3 and STAT3 after KDM3A overexpression were evaluated using western blotting. Upper panel: Representative western blot images; lower panel: Statistical analysis of western blot results (*n* ═ 3). All experiments were performed at least three times, and statistical analyses were conducted using Student's *t*-test. Significance levels are indicated as follows: *p < 0.05; **p < 0.01; ***p < 0.001, ns: no significant differences. Abbreviations: KDM3A: Lysine-specific demethylase 3A; H3K9me2: Histone H3 lysine 9 dimethylation; VIM: Vimentin; MMP-9/MMP9: Matrix metalloproteinase-9; pSTAT3: Phosphorylated STAT3; STAT3: Signal transducer and activator of transcription 3.

To elucidate the direct regulatory mechanism of KDM3A on EMT-related genes, we performed ChIP qPCR using an H3K9me2 antibody. Compared to control cells, we observed a significant enhancement in the enrichment of H3K9me2 at the promoters of VIM and MMP9 in KDM3A knockdown cells (Figure 4A, left panel). Importantly, KDM3A overexpression significantly reduced H3K9me2 occupancy at these promoters (Figure 4A, right panel), indicating direct epigenetic de-repression. In contrast, no significant H3K9me2 enrichment was detected at the *CDH1* (E-cadherin) promoter under either condition (Figure 4A), suggesting an alternative regulatory mechanism. Previous studies have indicated that KDM3A can activate the STAT3 pathway, which suppresses E-cadherin expression [22–25]. We investigated this possibility and found that KDM3A knockdown inhibited STAT3 and p-STAT3 (Y705) expression (Figure 4B), whereas KDM3A overexpression enhanced their levels (Figure 4C). In summary, these results demonstrate that *KDM3A* promotes EMT and invasion through distinct mechanisms: it directly reduces H3K9me2 at the *VIM* and *MMP9* promoters to activate their expression, while likely suppressing E-cadherin indirectly via activation of the STAT3 pathway.

### KDM3A overexpression enhances cell proliferation, migration, and invasion

**Figure 5.**
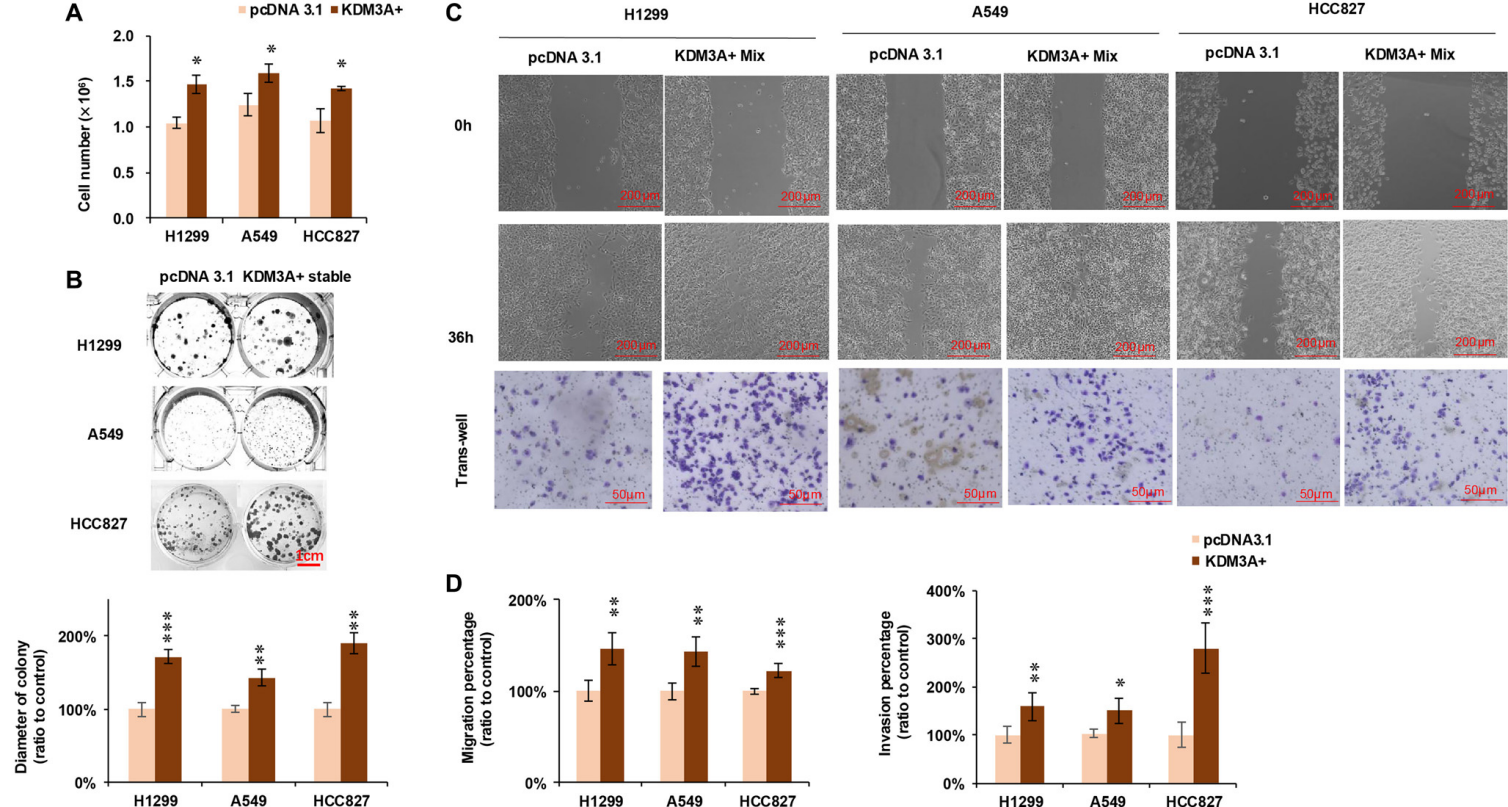
**Overexpression of KDM3A enhances cell proliferation, migration, invasion and colony formation.** (A) Quantification of cell numbers in H1299, A549, and HCC827 cell lines after 72 hours of KDM3A overexpression (*n* ═ 3). (B) Colony formation was enhanced in H1299, A549, and HCC827 following stable KDM3A overexpression. Lower panel: representative images of the cells; scale bar: 1 cm. Right panel: quantification of cell colony diameters, expressed as a ratio to the pcDNA 3.1 control group (*n* ═ 100). (C) Upper panel: Wound healing assay demonstrating the migratory capacity of H1299, A549, and HCC827 cells, revealing a significant increase in migration following KDM3A overexpression. Scale bar: 200 µm. Lower panel: representative images from the invasion assay, indicating a marked enhancement in invasive ability after KDM3A overexpression. Scale bar: 50 µm. (D) Quantification of migratory distance and the number of invaded cells (*n* ═ 5). All experiments were conducted at least three times, and statistical analysis was performed using the Student's *t*-test. *P < 0.05; **P < 0.01; ***P < 0.001. ns: no significant differences. Abbreviations: KDM3A: Lysine-specific demethylase 3A; H1299/A549/HCC827: NSCLC cell lines.

To further characterize the pro-tumorigenic role of KDM3A, we assessed its impact on proliferation, clonogenicity, migration, and invasion. Cell counting assays revealed that KDM3A overexpression significantly enhanced the proliferation of H1299, A549, and HCC827 cell lines (Figure 5A). Consistent with this, a colony formation assay demonstrated that KDM3A overexpression increased the colony-forming ability of all three cell lines compared to the control group (Figure 5B). Additionally, a 36-h wound healing assay revealed enhanced migration of cancer cells following KDM3A overexpression (Figure 5C, upper panel and Figure 5D, left panel). The transwell experiment also indicated increased invasive activity of cancer cells upon KDM3A overexpression (Figure 5C, lower panel and Figure 5D, right panel). These findings demonstrate that KDM3A overexpression drives NSCLC progression by enhancing key cancer hallmarks, including proliferation, clonogenicity, migration, and invasion.

### KDM3A knockdown reduces tumor formation *in vivo*

**Figure 6.**
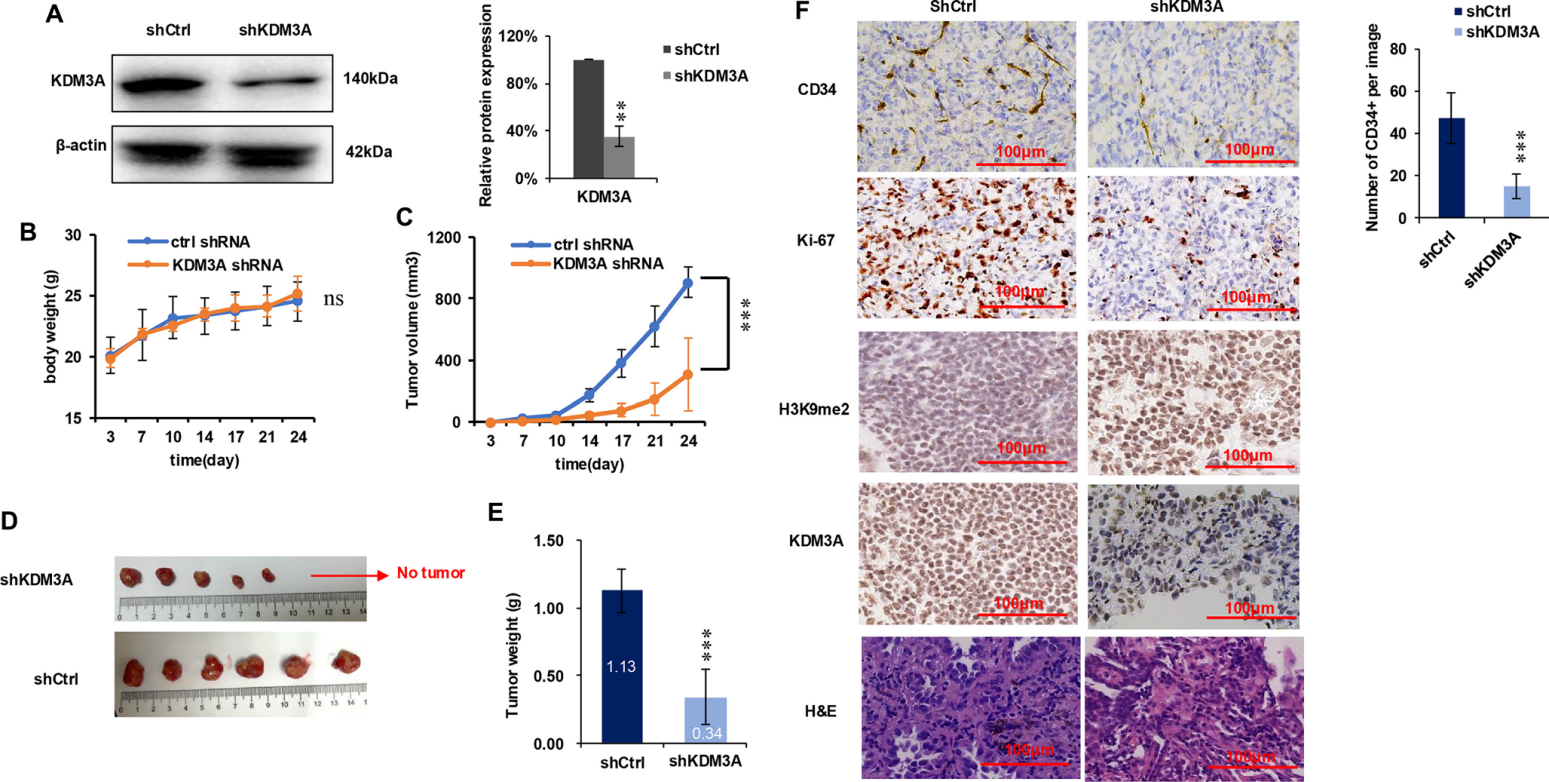
**Knockdown of KDM3A decreases tumor formation *in vivo*.** (A) KDM3A was stably knocked down in H1299 cells. Whole cell lysates were analyzed using Western blotting, with β-actin serving as a loading control. The left panel displays representative Western blot images, while the right panel presents statistical analysis of the Western blot results (*n* ═ 3). (B) No significant difference in body weight was observed between the two groups (*n* ═ 6, where *n* indicates the number of mice per group). (C) The KDM3A knockdown group exhibited a slower tumor growth rate compared to the control group. Tumor growth was monitored twice weekly, and tumor volume was calculated using the formula 0.5 × L × W^2^ (*n* ═ 6). (D) Images of tumor samples from the shCtrl and shKDM3A groups are shown. (E) Tumor weight quantification for the shCtrl and shKDM3A groups is presented (*n* ═ 6). (F) The left panel includes representative images of HE staining and immunohistochemical staining for KDM3A, H3K9me2, Ki67, and CD34 in tumor tissues, with a scale bar of 100 µm. The right panel shows microvessel quantification based on CD34 IHC staining (*n* ═ 12, where n represents the number of images analyzed for each group, with two images per mouse). All statistical analyses were conducted using the Student's *t*-test. Significance levels are indicated as follows: *p < 0.05; **p < 0.01; ***p < 0.001. ns: no significant differences. Abbreviations: KDM3A: Lysine-specific demethylase 3A; H1299: NSCLC cell line; H3K9me2: Histone H3 lysine 9 dimethylation; HE: Hematoxylin-eosin; Ki-67/KI67: Proliferation marker Ki-67.

To investigate the role of KDM3A in tumor growth *in vivo*, we established stable KDM3A shRNA knockdown H1299 cell lines using shRNA. As confirmed by Western blot analysis, KDM3A was stably knocked down in the shKDM3A group compared to the shCtrl group (Figure 6A, left and right panels). The body weights of the mice showed no significant difference between the shCtrl and shKDM3A groups (Figure 6B), indicating that KDM3A knockdown of H1299 cells did not cause systemic toxicity of mice. However, tumor growth was significantly inhibited upon KDM3A knockdown, as evidenced by reduced tumor volume and weight compared to the shCtrl group (Figure 6C–6E).

Furthermore, IHC analysis of the xenograft tumors revealed that the shKDM3A group exhibited decreased KDM3A expression, increased H3K9me2 levels, and reduced expression of the proliferation marker Ki-67 and the angiogenesis marker CD34 (Figure 6F). These results indicate that KDM3A knockdown suppresses tumor cell proliferation and angiogenesis *in vivo*. Collectively, these *in vivo* findings demonstrate that KDM3A knockdown significantly inhibits tumor growth, consistent with our *in vitro* observations.

### KDM3A as a potential therapeutic target for NSCLC

**Figure 7.**
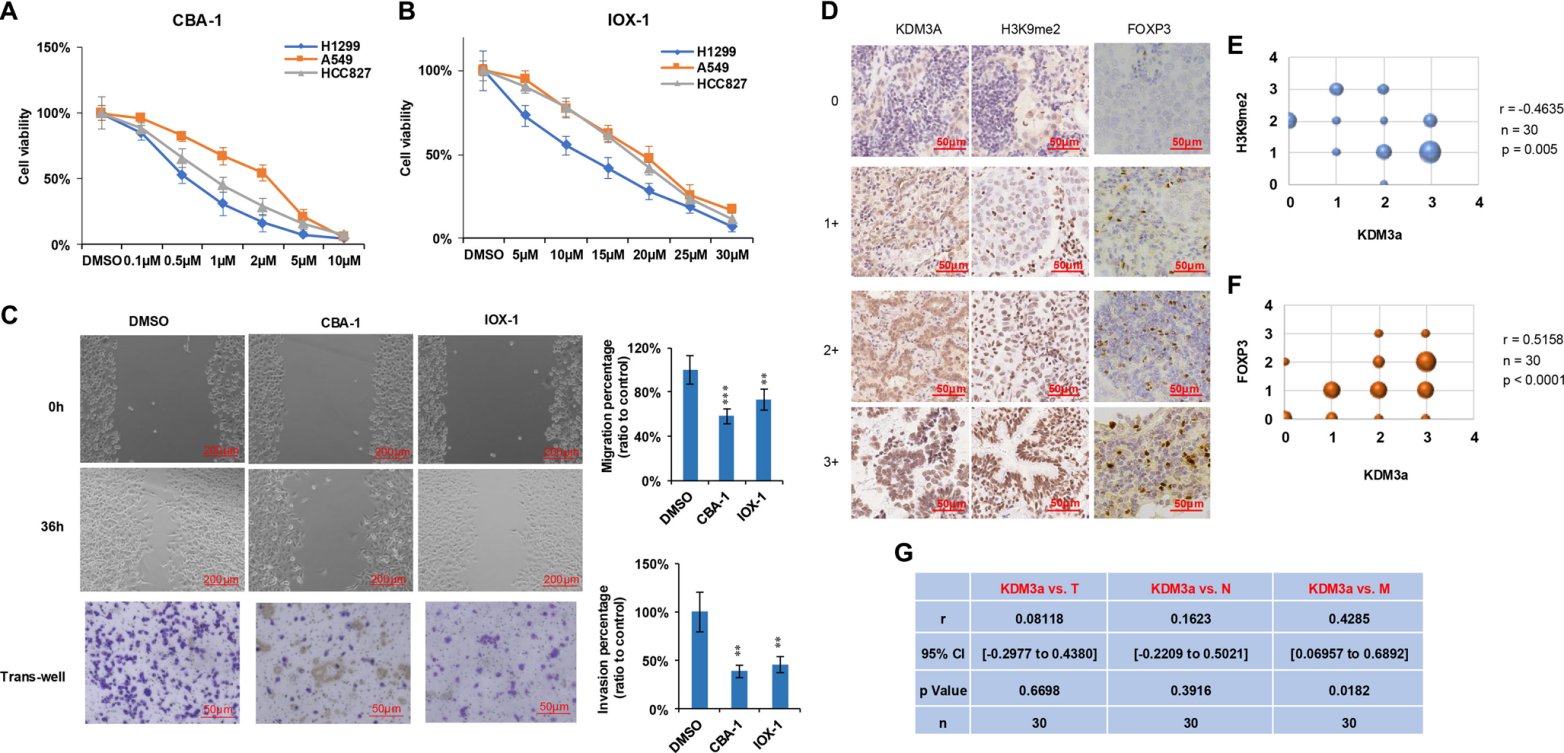
**KDM3A as a potential therapeutic target for NSCLC.** (A and B) The KDM3A inhibitors CBA-1 and IOX-1 significantly decreased cell viability in all three NSCLC cell lines (*n* ═ 5). (C) Both CBA-1 and IOX-1 inhibited the migration and invasion of H1299 cells. H1299 cells were treated with CBA-1 or IOX-1, followed by a wound healing assay to assess migratory ability and a transwell assay to evaluate invasive capability. Left panel: representative images of cells. Right panel: statistical analysis of migration distance and invasion percentage. Statistical analysis was performed using the Student's *t*-test (*n* ═ 5). (D) IHC scores for KDM3A, H3K9me2, and FOXP3 in clinical patient samples. Scale bar: 50 µm. (E) Spearman correlation between KDM3A and H3K9me2 IHC scores (*n* ═ 30). (F) Spearman correlation between KDM3A and FOXP3 IHC scores (*n* ═ 30). (G) Spearman correlation between KDM3A and TNM stage (*n* ═ 30). Significance levels are indicated as follows: *P < 0.05; **P < 0.01; ***P < 0.001. Abbreviations: KDM3A: Lysine-specific demethylase 3A; NSCLC: Non-small cell lung cancer; CBA-1: Small-molecule KDM inhibitor; H1299/A549/HCC827: NSCLC cell lines; H3K9me2: Histone H3 lysine 9 dimethylation.

To evaluate the therapeutic potential of targeting KDM3A in NSCLC, we treated H1299, A549, and HCC827 cell lines with two KDM3A inhibitors with limited selectivity, CBA-1 and IOX-1. Both compounds significantly reduced cell viability (Figure 7A and 7B) and potently suppressed migration and invasion in H1299 cells (Figure 7C). Surprisingly, when treating the cells with the two inhibitors in combination with cisplatin, no synergistic effect was observed (Figure S1A–S1B). In 30 primary NSCLC specimens, we stained for the expression of KDM3A, H3K9me2, and FOXP3 (Figure 7D) and assessed the relationship between KDM3A, H3K9me2, FOXP3+ Treg infiltration, and TNM stage. KDM3A expression exhibited a significant inverse correlation with H3K9me2 levels (r ═ −0.4635, *P* < 0.01; Figure 7E), consistent with its demethylase activity. Strikingly, KDM3A positively correlated with FOXP3 expression (*r* ═ 0.5158, *P* < 0.0001; Figure 7F), suggesting a potential role in Treg recruitment or expansion. Furthermore, elevated KDM3A levels were associated with metastasis (M stage) (*r* ═ 0.4285, *P* ═ 0.0182), supporting its pro-metastatic function (Figure 7G). Collectively, these findings indicate that pharmacological inhibition of KDM3A suppresses tumor growth and invasive potential, while its overexpression in clinical specimens correlates with metastatic progression and infiltration of immunosuppressive regulatory T cells.

## Discussion

This study thoroughly examined the role of the H3K9me2 demethylase KDM3A in the progression of NSCLC. Our findings establish KDM3A as a pivotal epigenetic regulator of epithelial–mesenchymal transition (EMT) and metastasis. Knockdown of KDM3A resulted in elevated global H3K9me2 levels, leading to the upregulation of the epithelial marker E-cadherin and the downregulation of mesenchymal markers such as Vimentin and MMP-9. This alteration suppressed cell proliferation, migration, and invasion. Conversely, overexpression of KDM3A decreased H3K9me2 levels, promoted a mesenchymal phenotype, and enhanced aggressive cellular behaviors. These *in vitro* results were corroborated *in vivo*, where KDM3A knockdown significantly inhibited tumor growth in xenograft models. Mechanistically, we demonstrated that KDM3A directly reduced H3K9me2 occupancy at the promoters of *VIM* and *MMP9*, thereby activating their transcription. Additionally, KDM3A downregulated E-cadherin expression through the activation of the STAT3 signaling pathway. Clinically, elevated KDM3A expression in NSCLC patient samples was inversely correlated with H3K9me2 levels and positively associated with metastasis and FOXP3+ Treg infiltration, highlighting its clinical significance.

As cancer advances, cells known as epithelial tumors may undergo EMT, thereby profoundly altering the characteristics of the tumor cells. This caused the disappearance of epithelial markers (i.e., E-cadherin), alterations in cell polarity and intercellular junctions, and a rise in mesenchymal indicators (i.e., Vimentin) [26]. Thus, with increasing disease severity, the expression of E-cadherin tends to decrease, while the expression of Vimentin tends to increase and can be used as a marker.

In this study, KDM3A knockdown led to increased H3K9me2 levels, elevated E-cadherin expression, and decreased expression of Vimentin and MMP-9. Subsequent analyses indicated that KDM3A knockdown and inhibition resulted in reduced tumor cell proliferation, migration, and invasion. In contrast, KDM3A overexpression decreased H3K9me2 levels, reduced E-cadherin expression, and increased Vimentin and MMP-9 levels. Experiments involving KDM3A overexpressing cells demonstrated a significant increase in tumor cell invasion, migration, and proliferation. Furthermore, the xenograft mouse model revealed that KDM3A knockdown significantly reduced tumor growth *in vivo*. Our findings indicate a correlation between KDM3A expression, H3K9me2 levels, metastasis stage, and Treg infiltration in patient samples.

Our findings suggest that the regulation of invasion-related genes by the H3K9 methylation regulator KDM3A significantly influences the proliferation, migration, and invasion of NSCLC. The data strongly support KDM3A’s role in enhancing NSCLC cell proliferation and invasion through H3K9 demethylation of *VIM* and *MMP-9*, as well as the regulation of E-cadherin. Therefore, KDM3A is critical in the regulation of EMT in NSCLC.

In addition to its effects on the epigenetic landscape, particularly H3K9me2, KDM3A exhibits other epigenetic functions. *KDM3A* directly binds to the *FOXP3* promoter, stimulating *FOXP3* transcription and inducing the release of downstream inhibitory cytokines (TGF-β1, IL-35, and HO-1) associated with FOXP3, which ultimately facilitates immune escape in lung adenocarcinoma [27]. Our study found a significant positive correlation between KDM3A and FOXP3 expression in clinical NSCLC patient samples, prompting further investigation into KDM3A’s role in immune infiltration in NSCLC. KDM3A also regulates epidermal growth factor receptor (*EGFR*) expression through kruppel-like factor 5 (*KLF5*) and SMAD family member 4 (*SMAD4*) [28]. Moreover, an epigenetic network involving Let-7d, KDM3A, and ENO2 has been identified in the pathogenesis of preeclampsia, where Let-7d functions as a miRNA that inhibits preeclampsia development by downregulating KDM3A expression and promoting ENO2 methylation, thereby inhibiting trophoblast development and inducing apoptosis [29].

Research indicates that histone methylation status is associated with cell proliferation, survival, differentiation, and gene expression in various human diseases [30, 31]. Multiple lysine methyltransferases (KMTs) facilitate the methylation of lysine residues, most of which are reversible and mediated by lysine-specific demethylases (KDMs) [32]. Understanding cancer’s origins necessitates unraveling the dysregulated regulators of histone lysine methylation and exploring their underlying mechanisms. Our systematic examination of the H3K9 regulator KDM3A in NSCLC revealed that it promotes cell proliferation and invasion by demethylating H3K9me2 of *VIM* and *MMP-9*. Consistent with our findings, it has been reported that aberrant H3K9 methylation leads to the transcriptional repression of various tumor suppressor genes in cancer cells [33], affecting the capacity of these cells to proliferate, spread, and infiltrate, as observed in malignancies such as colorectal cancer [34], melanoma [35], prostate cancer [36], gastric cancer [37], breast cancer [38], lung adenocarcinoma [39], and triple-negative breast cancer [40]. Aberrant H3K9 methylation is linked to enhanced proliferation, migration, and invasiveness in these tumors. Besides, *KDM3A* was identified to demethylate H3K9me2 at the promoter of specificity protein 1 (SP1) and to increase SP1 transcription in osteosarcoma and pancreatic cancer in a study by Wang et al. Restoring SP1 prevented cancer cells from proliferating and spreading and restored glycolytic flow to cells suppressed by KDM3A knockdown. As a result, KDM3A knockdown dramatically reduced the development of tumors *in vivo* and *in vitro* and inhibited the aerobic glycolysis of cancer cells [41, 42].

Additionally, KDM3A has been linked with promoting invasion and migration in NSCLC [43]. Moreover, KDM3A overexpression was identified in colorectal cancer specimens, and KDM3A knockdown reduced MMP-9 expression and enzyme activity. This knockdown inhibited colorectal cancer cell migration, invasion, and metastasis [44]. MMP-9 belongs to the MMP family and is a zinc-dependent peptidase expressed by many cellular [45]. E-cadherin inhibits cell migration and dissemination by mediating cell-cell adhesion dependent homologous interactions via Ca2+ [21]. Matrix metalloproteinases (MMPs) are zinc-dependent proteases with specific protein hydrolysis effects. MMP-9-mediated collagen degradation promoted cancer cell invasion and metastasis [46, 47]. The downregulation of E-cadherin diminishes cell–cell adhesion, enhancing cell viability and β-catenin activation, thereby promoting malignancy [48]. Our study similarly demonstrated that KDM3A knockdown in three lung cancer cell lines reduced the expression of invasion-related genes such as Vimentin and MMP-9 while increasing E-cadherin expression, ultimately inhibiting cancer cell migration. Conversely, KDM3A overexpression enhanced cell migration, invasion, and colony formation, as well as the expression of EMT-related genes in NSCLC cells.

Numerous studies have investigated the effects of KDM3A on lung cancer; however, comprehensive analyses focusing on the epigenetic effects and specific downstream targets of KDM3A and H3K9me2 in lung cancer remain insufficient. This study comprehensively examines the alterations in H3K9me2 levels following KDM3A overexpression and knockdown, along with their impacts on the invasion of NSCLC cells. Utilizing ChIP qPCR, we identified that *KDM3A* directly modulates H3K9me2 occupancy at the promoters of *VIM* and *MMP-9*, with knockdown resulting in increased occupancy and overexpression leading to decreased occupancy, thereby regulating the expression of VIM and MMP-9. Furthermore, KDM3A downregulated E-cadherin expression via the p-STAT3 signaling pathway. Building on prior research [23], we conclude that *KDM3A* indirectly regulates *CDH1* expression, potentially through the STAT3 signaling pathway. The integration of both *in vitro* and *in vivo* experiments, as well as clinical samples, enhances the robustness of our findings. *In vivo* studies provide advantages such as precise control of exposure conditions, simultaneous measurement of multiple effects, assessment of host characteristics, and evaluation of underlying mechanisms. Animal models have become essential tools for elucidating cancer mechanisms. Several experiments have explored the influence of KDM3A on NSCLC through validation in *in vivo* models. One study demonstrated that in tests with nude mice, microRNA-449a targeted the KDM3A/hypoxia-inducible factor (HIF-1α) axis to inhibit lung cancer progression [49]. Additionally, researchers have shown that KDM3A expression significantly affected the reduction of *EGFR* in NSCLC with *EGFR* mutations. Our study employed three cell lines harboring RAS or EGFR mutations, all of which exhibited significant migratory and invasive responses upon KDM3A modulation, indicating that the KDM3A signaling pathway in NSCLC operates independently of the cancer cell’s genetic background.

Lung cancer progression is regulated by multiple mechanisms mediated by key regulatory factors. The anticancer effects of the inhibitor BIX have been linked to the downregulation of the phosphorylated BCKD E1alpha subunit (BCKDHA) by KDM3A [1]. Authors xenografted H1299 cells into nude mice and investigated the effects of the long-chain non-coding RNA small nucleolar RNA host gene 4 (SNHG4) on NSCLC. Their findings indicated that KDM3A inhibits p21, and that RNA SNHG4 contributes to the oncogenic effects associated with NSCLC progression through the regulation of KDM3A, corroborating our results [43]. In our study, we conducted animal experiments by introducing KDM3A shRNA into cells, followed by transplanting the KDM3A stable knockdown cells into mice. We assessed the impact of KDM3A knockdown on tumor growth by comparing tumor volume and weight between the knockdown and control groups. The experimental data indicated that tumor weight and volume in the KDM3A knockdown group were significantly lower than those in the control group. This outcome, combined with our previous *in vitro* experiments, confirms that KDM3A knockdown substantially inhibits tumor growth in both *in vitro* and *in vivo* settings.

In studies focused on NSCLC, abnormally elevated KDM3A expression has been observed in malignant tissues, and KDM3A knockdown has been shown to inhibit critical cancer cell functions, underscoring its clinical significance [50]. Nonetheless, despite the ongoing development of KDM inhibitors, the compounds reported thus far lack selectivity for the KDM3 subfamily or its specific isoforms. This limitation is largely due to the fact that most KDM3 crystal structures remain unresolved, and existing inhibitors often target catalytic mechanisms that are common to a wide range of KDMs [51]. In this study, we utilized two KDM3A inhibitors with limited selectivity, CBA-1 and IOX-1, and observed that treatment significantly inhibited the viability, migratory, and invasive capabilities of NSCLC cells. However, we acknowledge significant limitations: the currently available KDM3A inhibitors lack sufficient specificity. CBA-1 exhibits additional anti-Wnt activity [52], while IOX-1 broadly targets multiple KDMs [51]. These off-target effects may complicate the interpretation of KDM3A’s specific role in our models. The development of more potent and highly selective KDM3A inhibitors will be essential for future therapeutic investigations.

This study elucidates the pivotal role of KDM3A in facilitating the proliferation and invasion of NSCLC cells through H3K9 demethylation. Analysis of clinical samples further correlates elevated KDM3A expression with metastasis and immune infiltration. These findings underscore KDM3A’s consistent involvement in NSCLC progression across both experimental and clinical contexts, highlighting its potential as a prognostic biomarker and therapeutic target. Targeting KDM3A may enhance precision medicine approaches and pave the way for novel epigenetic treatments for NSCLC.

This study has several limitations. First, while we identified that KDM3A downregulates H3K9me2 in NSCLC, we did not thoroughly investigate whether its knockdown or overexpression influences activity of other KDMs. Second, we found that *KDM3A* regulates *VIM* (vimentin) and *MMP-9* through removing promoter occupancy of H3K9me2. Unfortunately, we could not find a ChIP grade antibody for KDM3A and could not get the KDM3A occupancy data in the EMT genes. Through literature consultation and preliminary experiments, we speculated *KDM3A* regulated *CDH1* through STAT3 signaling pathway, this need to be further validated by rescue experiment. Third, since KDM3A expression induces a global change in H3K9me2 levels, its potential regulation of tumor suppressor genes needs to be verified in subsequent studies. Fourth, regarding the correlation between KDM3A and immune infiltration, we only observed this association in patient tissues. Although previous studies have shown that *KDM3A* regulates *FOXP3* transcription in lung cancer [27], further validation in immunocompetent mouse model is necessary to definitively investigate the role of KDM3A in immune infiltration.

Although our evidence supports a direct association between KDM3A and genes governing cell invasion, additional research is necessary to elucidate the underlying regulatory mechanisms. A comprehensive understanding of the epigenetic regulation mediated by KDM3A is vital and may involve coordinated interactions between histone modifications and nucleosome structure.

## Conclusion

In conclusion, our study establishes KDM3A as a key epigenetic driver of NSCLC progression, promoting tumor growth and invasion through the regulation of H3K9 methylation. This work positions KDM3A as a compelling therapeutic target for the development of innovative treatment strategies against NSCLC.

## Supplemental data

**Figure S1.**
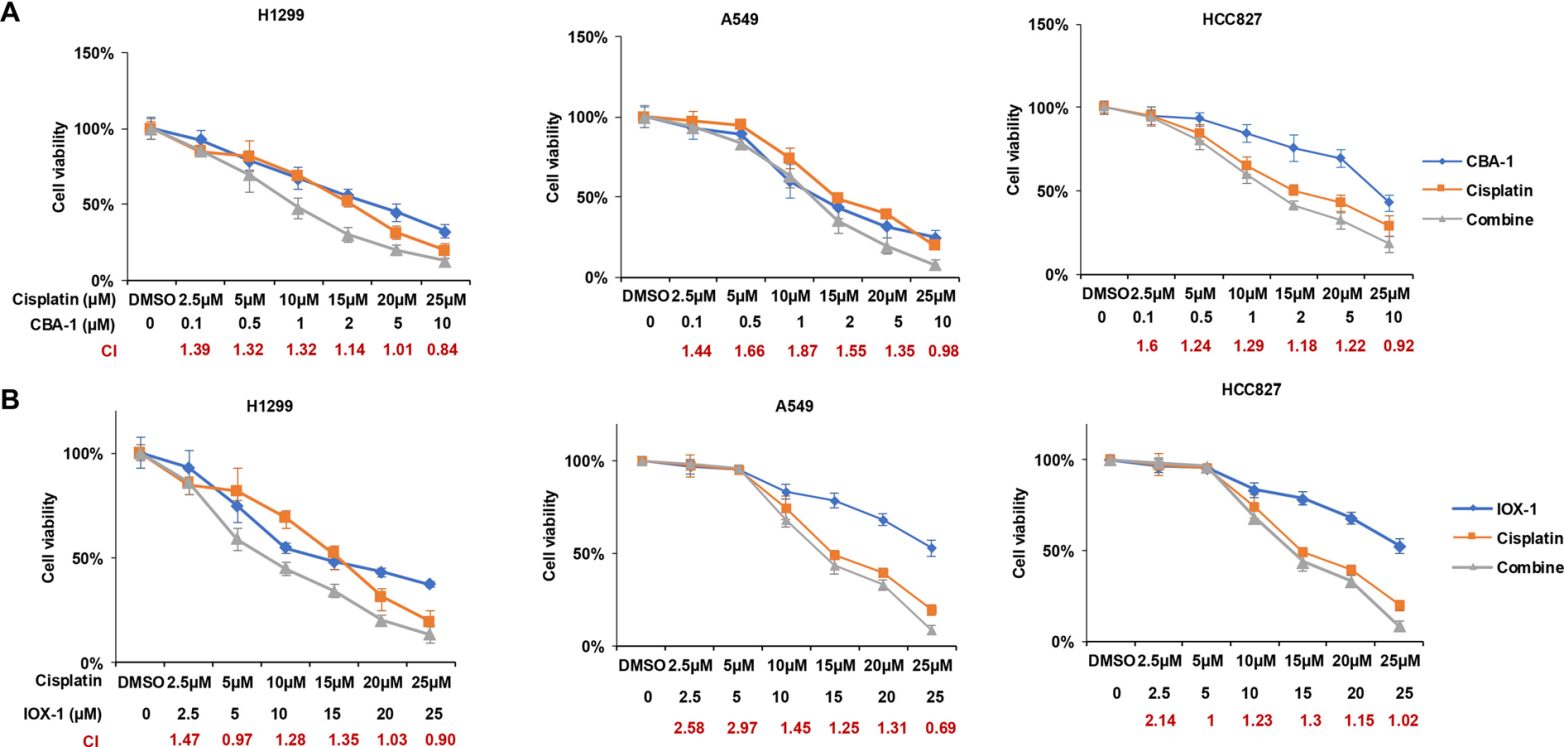
**The combination therapy of KDM3A and cisplatin treatment demonstrated no synergistic inhibition for NSCLC *in vitro*.** (A) Cell viability of the three cell lines following treatment with CBA-1, cisplatin alone, or the combination at the indicated concentrations (*n* ═ 5); (B) Cell viability of the three cell lines following treatment with IOX-1, cisplatin alone, or the combination at the indicated concentrations (*n* ═ 5). Abbreviations: KDM3A: Lysine-specific demethylase 3A; NSCLC: Non-small cell lung cancer; CBA-1: Small-molecule KDM inhibitor; IOX-1: Broad-spectrum KDM inhibitor; H1299/A549/HCC827: NSCLC cell lines.

## Data Availability

The original data for statistical analysis are included in the “original data.xls” file, which contains tables of body weight and tumor measurements for each mouse.
